# Toward the calibration of serological assays using sera collected from cattle and sheep following a single dose of foot-and-mouth disease vaccine

**DOI:** 10.14202/vetworld.2022.524-530

**Published:** 2022-02-28

**Authors:** Aiken S. Karabassova, Akhmetzhan A. Sultanov, Meruyert A. Saduakassova, Donald P. King, Anna B. Ludi, Clare F. J. Browning, Ginette Wilsden

**Affiliations:** 1 Department for Epizootological Monitoring and Risks Assessment of Animal Viral Diseases, The Kazakh Scientific Research Veterinary Institute, 223 Raimbek Avenue, Almaty, Kazakhstan; 2 The Pirbright Institute, Ash Road, Pirbright, Woking, Surrey GU24 0NF, United Kingdom

**Keywords:** cattle, enzyme-linked immunoassay, foot-and-mouth disease, immunogenicity, post-vaccination monitoring, sheep, virus neutralization test

## Abstract

**Background and Aim:**

Serological assays are widely used to monitor the performance of foot-and-mouth disease (FMD) vaccines to estimate vaccination coverage and to ensure that vaccinated animals generate adequate immune responses. This study aimed to measure the FMD virus (FMDV)-specific responses in cattle and sheep after a single dose of a trivalent FMD vaccine containing serotypes A, O, and Asia-1, and to use these sera to calibrate virus neutralization tests (VNTs) and serotype-specific serological enzyme-linked immunoassays (ELISAs) that can measure post-vaccination responses.

**Materials and Methods:**

Sera were collected from cattle (n=10) and sheep (n=10) on 0, 21, and 56 days after immunization with an imported aqueous formulated FMD vaccine. These samples were tested by VNT using field FMDV isolates that are representative of the epidemiological risks in Central Asia (A/ASIA/Iran-05, A/ASIA/GVII, O/ME-SA/Ind-2001, O/SEA/Mya-98, O/ME-SA/PanAsia, and Asia-1 Shamir). Heterologous VNT antibody responses were compared to those measured using commercial FMDV-specific ELISAs for serotypes O, A, and Asia 1.

**Results:**

Administration of the FMD vaccine increased FMDV-specific antibody titers for both species in sera collected on day 21, but these elevated titers were short-lived and were decreased by day 56.

**Conclusion:**

These results highlight the short duration of immunity with a single dose of this aqueous vaccine and motivate further studies to assess immune responses in cattle and small ruminants after a two-dose course vaccination schedule. Further comparative data for VNT and serotype-specific ELISAs are needed to define cutoffs that can be used to monitor post-vaccination immune responses in low-containment laboratories where it is not possible to handle live FMDVs.

## Introduction

Foot-and-mouth disease (FMD) is caused by a picornavirus (FMD virus [FMDV]: Family: *Picornaviridae*; genus: *Aphthovirus*) which is endemic across Africa, many countries in Asia and parts of South America. FMD is a major constraint to international trade, and for this reason, the prevention and control of the disease is a high priority that shapes animal health policies in many countries. Every year, more than 2 billion doses of FMD vaccines are used worldwide (i) to control FMD outbreaks and (ii) for prophylactic purposes to limit disease incursions and spread of the virus in endemic regions [1]. Most FMD vaccines comprise inactivated cell culture-grown FMDVs formulated with oil-based adjuvants [typically Montanide^®^ ISA25 or ISA206 (SEPPIC, Paris, France)] or water-based adjuvants (typically aluminum hydroxide [Al(OH)_3_] and saponin) [2]. The effectiveness of FMD vaccines is limited by their relatively short duration of immunity and dependence on a cold chain and antigenic diversity among field viruses [3]. Despite these issues, FMD vaccines have been used with other zoosanitary measures to successfully eradicate FMD from Europe and most of South America [4,5].

**Figure-1:**
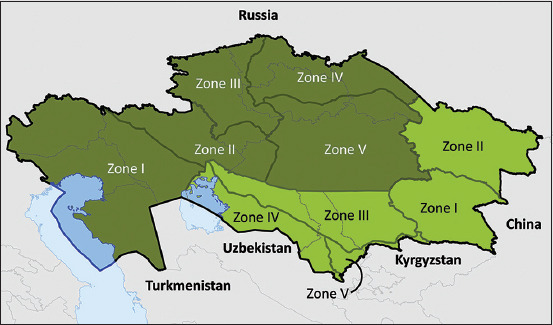
Current foot-and-mouth disease (FMD) zones for Kazakhstan. Dark green areas represent FMD-free zones where vaccination is not practiced and light green areas are areas that are FMD-free zones where vaccination is practiced [Source: https://www.oie.int/en/disease/foot-and-mouth-disease/#ui-id-2].

Kazakhstan is a large country in Central Asia, bordering the Russian Federation to the north and west, China in the east, and Kyrgyzstan, Uzbekistan, and Turkmenistan in the south. According to the Kazakh Statistical Information Service, in 2020, there were about 8.1 million head of cattle and 71.7 million head of sheep in the country that are mostly reared on the vast grazing lands of the country. FMD was reported during 1955-2013 in Kazakhstan [6]. These outbreaks were associated with serotypes O and A of FMDV where the incidence was usually highest in cattle, followed by sheep [6]. The last case of FMD was recorded in 2013 in the East Kazakhstan region and since mid-2013, a high-potency (>6 50% protective dose [PD50]) vaccine, purified from non-structural proteins, containing FMDV antigens relevant for Kazakhstan has been used in the country. In 2017, the Republic of Kazakhstan received World Organization for Animal Health (OIE) recognition for its status as FMD free with vaccination for a zone comprising five regions of the country: Almaty, East Kazakhstan, Zhambyl, Kyzylorda, and South Kazakhstan. In the remainder of the country to the north of this zone, there are five zones (Figure-1) free from FMD where vaccination is not practiced.

In the vaccination zones, the use of imported vaccines continues to be the main tactic to protect animals and limit the potential spread of FMD from neighboring countries to the south and east of Kazakhstan where different FMD viral lineages are present such as O/ME-SA/PanAsia, O/ME-SA/PanAsia-2, O/SEA/Mya-98, A/ASIA/Iran-05, A/ASIA/G-VII, A/ASIA/Sea-97, and serotype Asia 1 [7-10]. Young cattle are immunized from 4 months of age while sheep and goats are vaccinated from 3 months of age and both species are revaccinated every 3 months until they reach 18 months. Adult animals are vaccinated every 6 months. Considering the importance of vaccines in FMD control, the Food and Agriculture Organization (FAO) and OIE have recently published the FMD vaccination and post-vaccine monitoring (PVM) guidelines to provide direction on standards and methods that are recommended to assess the performance of FMD vaccines [11]. As part of this work, immunogenicity studies are recommended to provide empirical data to evaluate immune responses in host species.

This study aimed to apply the PVM guidelines by assessing the immunogenicity of an imported FMD vaccine produced by the Federal Centre for Animal Health (FGBI) “ARRIAH” (Vladimir, Russia) in cattle and sheep. Serotype-specific antibodies for serotypes A, O, and Asia-1 were measured using virus neutralization test (VNT) and commercial enzyme-linked immunoassay (ELISA) kits to allow comparison between these methods to monitor post-vaccination responses.

## Materials and Methods

### Ethical approval

The study was approved by local ethical commission (protocol no. 16/18). All animal handling procedures (in cattle and sheep) were undertaken according to legislative requirements.

### Study period and location

The study was conducted October 25 to December 31, 2019. The study was conducted on two commercial farms in the Talgar District of the Almaty region, which has had the status of FMD free with vaccination since 2017, and where serological monitoring for NSP-specific antibodies had not revealed evidence of circulating FMDV. The samples were processed at the Virology Laboratory of KazSRVI (LLP).

### Animals

Female cattle and sheep (10 animals from each species) with no previous history of FMD vaccination were selected for this study. These animals were brought from the FMD-free zone without vaccination in North Kazakhstan to the FMD-free zone with vaccination and were kept in quarantine for 30 days before the onset of the experimental work. The average age of the animals was 2.3 years for cattle and 1.8 years for sheep, respectively.

### FMD vaccine and vaccination

The trivalent vaccine used in this study was manufactured at the FGBI “ARRIAH” and contained the following vaccine strains A/Iran-05, A/Sea-97, O/PanAsia, O/PanAsia-2, and Asia-1/Shamir (providing at least six PD50 for each valency). The inactivated vaccine contained Al (OH)_3_ and saponin as adjuvants with the production date of June 2018 and was valid for use until December 2019. The dose was administered in accordance with the instructions of the vaccine manufacturer, with cattle receiving 3 ml of vaccine subcutaneously in the region of the middle third of the neck and sheep vaccinated with a 1 ml dose given subcutaneously in the inner thigh. Before use, the vaccine was stored in the dark at a temperature of 2-8°C.

### Blood sampling

Blood samples (n=20) were collected from all animals before inclusion in the study. All blood samples were taken by venipuncture from the jugular vein. The serum samples were tested and found to be negative for non-structural protein-specific antibodies using the Bionote NSP kit (Hwaseong-si, Gyeonggi-do, South Korea). All animals were identified separately through ear tags to ensure accurate observation and post-vaccination monitoring. During the study, serum samples were taken from the animals before the start of vaccination (day 0), 21, and 56 days after vaccination. Samples on ice were delivered to the virology laboratory of KazSRVI LLP (Almaty, Kazakhstan). On arrival at the laboratory, the sera were separated and frozen to −20°C until testing.

### Post-vaccination antibody testing

The samples were shipped on dry ice to the FAO World Reference Laboratory for FMD (WRLFMD) for testing by VNT and SP (specific antibodies)-ELISA for the detection of FMDV neutralizing and structural protein-specific antibodies. The VNT is the gold standard method used to assess the performance of FMD vaccines and in serosurveys to monitor the prevalence of different FMDV serotypes in livestock populations [11,12]. For VNT, heterologous titers were generated with the IB-RS-2 cell line according to the method outlined in the OIE Manual [12] using a virus dose of 100 tumor control dose 50 for field isolates representative of the epidemiological risks in Central Asia. The six FMDV isolates used included O/MOG/14/2017 (O/ME-SA/Ind-2001 lineage), O/MOG/4/2015 (O/SEA/Mya-98 lineage), O/MOG/13/2017 (O/ME-SA/PanAsia lineage), A/IRN/23/2018 (A/ASIA/Iran-05 lineage), A/IRN/25/2018 (A/ASIA/G-VII lineage), and Asia 1/Shamir. FMDV structural protein-specific antibodies were measured for the three FMDV serotypes (O, A, and Asia 1) using commercially available ELISA kits (PrioCHECK, Thermo Fisher, Waltham, MA, USA, and Istituto Zooprofilattico Sperimentale della Lombardia e dell’Emilia Romagna [IZSLER], Brescia, Italy) ELISA format according to the manufacturer’s instructions. These SP-ELISAs adopt a solid-phase competitive ELISA (SPCE) format where signal generated with an FMDV-specific monoclonal antibody is inhibited by FMDV-specific antibodies present in test sera.

## Results

### Baseline monitoring of animals

Daily assessment of all sheep and cattle showed that they remained in good health with no deterioration in appetite. There were no cases of suspected clinical FMD on either of the farms, and all animals were negative for non-structural protein-specific antibodies throughout the entire study period.

### Post-vaccination immune responses in cattle and sheep

**Figure-2:**
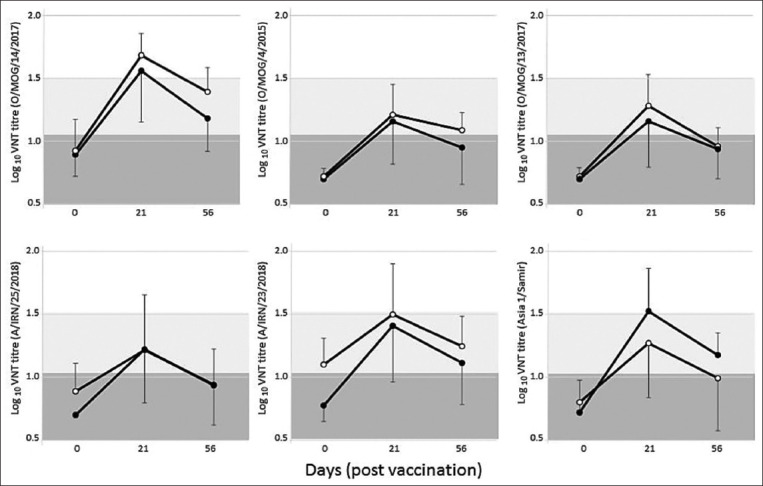
Post-vaccination heterologous virus neutralization test (VNT) responses in naïve cattle (•) and sheep (○) after a single dose of foot-and-mouth disease (FMD) vaccine. Data shown represent mean±standard deviation titers measured at three time points during the study with six different regional FMD virus reference antigens. Shaded boxes denote negative (dark gray: <1/11 titer) and inconclusive (light gray; >1/11 to <1/32 titer) VNT results.

Mean post-vaccination VNT titers for the cattle and sheep groups are shown in Figure-2, where FMDV-specific antibody titers measured on 21 days post-vaccination (DPV) were increased compared to sera collected before vaccination (day 0).

The highest heterologous VN titers on 21 DPV were measured for the O/MOG/14/2017 isolate (representing the O/ME-SA/Ind-2001 FMDV lineage) where mean titers were log_10_ 1.55±0.41 and log_10_ 1.68±0.17 for cattle and sheep, respectively. In contrast, the lowest 21-day post-vaccination titers were obtained for the O/MOG/4/2015 isolate where mean measured titers were log_10_ 1.16±0.34 and log_10_ 1.21±0.24 for cattle and sheep, respectively. Heterologous protective titers are not available from challenge studies for the vaccine components present in the studied vaccine. In the absence of specifically validated cutoffs, VNT titers greater or equal to log_10_ 1.5 were considered as an indicator of a minimum level of protection. This was the average titer associated with 50% protection comparing VNT titers obtained at WRLFMD, for serotypes O, A, and Asia 1, to animal protection test outcomes [13]. Based on this value, the greatest proportion of titers that exceeded this threshold on 21 DPV was generated with the O/ME-SA/Ind-2001 antigen (O/MOG/14/2017) where 6/10 and 9/10 were greater than log_10_ 5 for cattle and sheep, respectively (Table-1). In contrast, the O/ME-SA/PanAsia antigen (O/MOG/13/2017) only generated titers that exceeded this threshold for a single serum from a cow and three sheep. For all FMDV antigens, the heterologous titers measured on 56 DPV were reduced, highlighting that these FMDV-specific responses were short-lived after a single dose of this FMD vaccine.

**Table 1:** Proportion of individual sera with heterologous titers>log_10_ 1.5.

Types	Subtypes	Cattle		Sheep	
VNT antigen	FMD viral lineage	21 DPV	56 DPV	21 DPV	56 DPV
O/MOG/14/2017	O/ME-SA/Ind-2001d	6/10	1/10	9/10	4/10
O/MOG/4/2015	O/SEA/Mya-98	2/10	1/10	2/10	0/10
O/MOG/13/2017	O/ME-SA/PanAsia	1/10	0/10	3/10	0/10
A/IRN/23/2018	A/ASIA/Iran-05	5/10	2/10	6/10	4/10
A/IRN/25/2018	A/ASIA/G-VII	3/10	1/10	3/10	1/10
Asia 1 (Shamir)	Asia 1	5/10	3/10	5/10	0/10

DPV=Days post-vaccination, VNT=Virus neutralization test, FMD=Foot-and-mouth disease

### Correlation between VNT and ELISA methods

**Figure-3:**
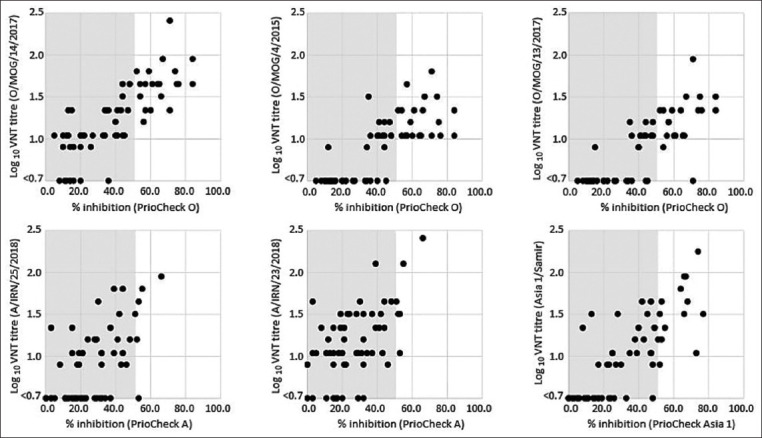
Pairwise comparison between enzyme-linked immunoassay (ELISA) responses (PrioCHECK) and heterologous virus neutralization test titers measured against six regional foot-and-mouth disease virus reference antigens. Gray areas denote negative ELISA values that are below the 50% inhibition cutoff for these tests.

**Figure-4:**
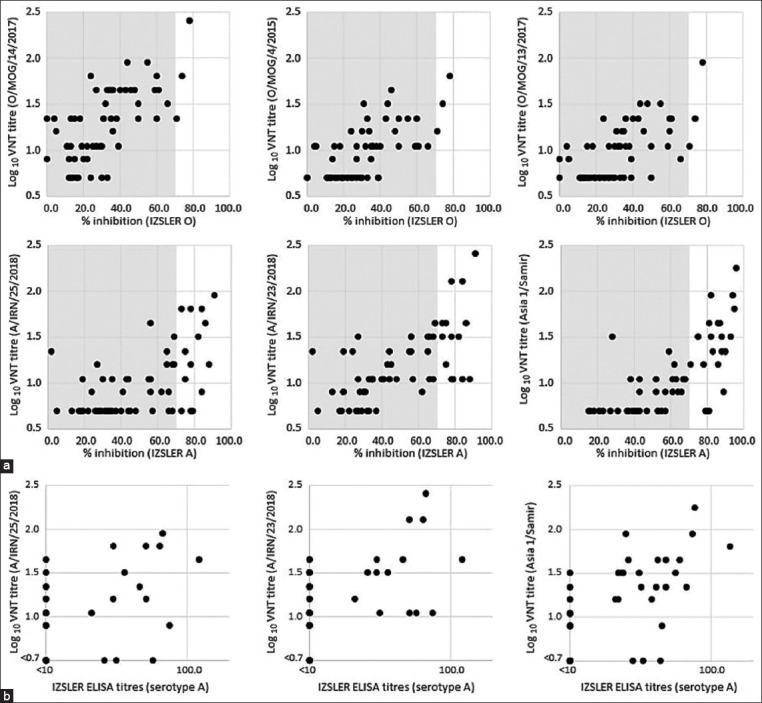
(a) Pairwise comparison between enzyme-linked immunoassay (ELISA) responses (Istituto Zooprofilattico Sperimentale della Lombardia e dell’Emilia Romagna) and heterologous virus neutralization test titers measured against six regional foot-and-mouth disease virus reference antigens. Gray areas denote negative ELISA values that are below the 70% inhibition cutoff for these tests. Calculated antibody titers for serotypes A and Asia 1 are shown in B.

Figures-3 and 4a display pairwise comparative data for the VNT results and raw percent inhibition (PI) values generated with the PrioCHECK and IZSLER SP ELISA kits where data were generated at a single dilution (1:10) for the sera. For the IZSLER SP assays, testing was also performed at different dilutions to determine a quantitative antibody titer. These calculated values are shown for serotypes A and Asia 1 in Figure-4b. Corresponding data for serotype O are not shown since only a very small proportion of the sera generated measurable titers with the serotype O test, where calculated titers >1/10 were only present with 3/30 and 0/30 of the cattle and sheep sera, respectively.

## Discussion

This study adopted an approach to determine the immunogenicity of an FMD vaccine in cattle and sheep and used VNT and ELISA methods to assess the serological responses in the vaccinated animals. This vaccine contained five component vaccine strains: Two for serotype O, two for serotype A, and one for serotype Asia 1. Rather than relying on homologous serological measurements (i.e., against the vaccine strains), this study employed field isolates representing virus lineages circulating in the Central Asian neighborhood to provide a measure of heterologous responses to FMDVs that pose great epidemiological risks. This approach using regional reference antigens for VNT has a number of advantages highlighted in a recent review [14], including the possibility to directly compare the immunogenicity of FMD vaccines from different suppliers and with different serotype compositions. A similar approach has been adopted recently for FMD vaccination studies undertaken in Mongolia to evaluate post-vaccination responses with aqueous and oil formulated vaccines in cattle, sheep, and camels [15], where the same three serotype O antigens as used in this study were chosen to represent O/ME-SA/Ind-2001, O/ME-SA/PanAsia, and O/SEA/Mya-98 lineages.

This report describes an initial study focused on a single-dose vaccination protocol using an aqueous FMD vaccine used in Kazakhstan. A 6 PD50 vaccine, which equates to a greater than 80% probability of protection, is expected to generate an average homologous log10 titer of greater than or equal to ~1.8 at ~3 weeks after vaccination for serotypes O, A, and Asia 1, using the WRLFMD VNT [13]. However, as the antigenic match between the vaccine and field viruses studied is unknown, a cutoff of 1.5 was selected, equating to a minimum level of protection (50% homologous protection; [13]). Elevated FMDV-specific titers were measured on 21 DPV for all three FMDV serotypes present in the vaccine. However, increases in heterologous FMDV-specific titers were modest and short-lived since samples collected on 56 DPV were lower than those measured on 21 DPV. A recent study undertaken in Mongolia using an FMD vaccine from the same supplier as used in Kazakhstan reports heterologous antibody results that were higher and exhibited a longer duration of response compared to the results described in this paper [15]. However, the specific composition of the vaccines used in these two studies was not equivalent; where five different FMDV antigens (A/Iran-05, A/Sea-97, O/PanAsia, O/PanAsia-2, and Asia-1/Shamir) were included in the vaccine used in Kazakhstan, compared to only two FMDV components (O/ME-SA/PanAsia and A/ASIA/Sea-97) for the vaccine in the Mongolian study. These data highlight the value of performing small-scale immunogenicity studies to support national FMD vaccine campaigns and reinforce (i) the importance of regular FMD vaccination in susceptible populations to ensure that adequate protective titers are elicited in the vaccinated animals and (ii) that vaccination is complemented by other zoosanitary measures [16,17].

VNT is widely used as an approach to measure FMDV-specific antibody responses and this method is recommended by the OIE for use to define the immune status of individual animals and populations [18]. However, since VNT requires the handling of live FMDV and cell culture work, it is only employed within a relatively small group of specialized high-containment laboratories. A central aim of this study was to use the post-vaccination sera to correlate VNT titers to corresponding measurements from commercial SP ELISA kits to provide simple post-vaccination metrics for the different serotypes that can be widely used by regional and field laboratories. However, it is important to consider that there is only a narrow range for the linear relationship between the raw PI values of the SP-ELISAs and VNT (as shown in Figures-3 and 4a). Therefore, if fully quantitative data are required, serum dilutions should be used in the SP-ELISAs to calculate antibody titers. Figure-4b displays comparative data for the VNT and IZSLER SP-ELISAs where results for two serotypes A antigens and serotype Asia 1 antigen are weakly correlated between the two test formats (R^2^ values of 0.3039, 0.2677, and 0.5077 for A/IRN/25/2018, A/IRN/23/2018, and Asia1/Shamir antigens, respectively). Unfortunately, the low number of weak to strongly positive sera in the study reduces the confidence in these comparative assessments, but an example of how these simple cutoffs might be established is provided by the data for the O/MOG/14/2017 isolate where a “protective” titer of 1.5 log_10_ equates to a 50% PI value on the PrioCHECK SP ELISA, at a sensitivity of 80.0% and a specificity of 90.0%.

This simple and relatively inexpensive study highlights some obvious gaps in empirical data that would improve confidence in the use of this and other vaccines to elicit protect responses in cattle and sheep. For example, the use of generic indirect serological cutoffs (either measured by VNT or SP-ELISA) could be enhanced by the provision of sera from challenge studies of the vaccine by the commercial supplier. Alternatively, sera from routine vaccine batch testing that has been correlated to protective responses by the manufacturer would also help to validate this approach. Although further work is required to improve the relationship between VNT and SP-ELISA (and ultimate protection in the host species), this study provides a framework for how this work can be undertaken.

## Conclusion

These data indicate that the FMD vaccine used here only elicited modest responses after primary vaccination. Therefore, further work is needed to investigate the impact of subsequent doses of this vaccine with the collection of sera over longer time periods to assess the waxing and waning of immunity in cattle and sheep. Analyses of additional sera representing enhanced immune responses after the second dose of vaccine will also improve our confidence in the correlation between VNT and SP-ELISA measurements.

## Authors’ Contributions

ASK: Concept and design of the study, data acquisition, analysis and interpretation of data, writing the article, and critical review of the manuscript. AAS: Concept and design of the study and critical review of the manuscript. MAS: Concept and design of the study, data acquisition, and critical review of the manuscript. DPK and ABL: Concept and design of the study, analysis and interpretation of data, writing and critical review of the manuscript. CB and GW: Data acquisition and critical review of the manuscript. All authors read and approved the final manuscript.
